# *In vitro* Models of Neurodegenerative Diseases

**DOI:** 10.3389/fcell.2020.00328

**Published:** 2020-05-13

**Authors:** Anna Slanzi, Giulia Iannoto, Barbara Rossi, Elena Zenaro, Gabriela Constantin

**Affiliations:** ^1^Department of Medicine, University of Verona, Verona, Italy; ^2^Center for Biomedical Computing (CBMC), University of Verona, Verona, Italy

**Keywords:** neurodegenerative diseases, *in vitro* models, three-dimensional culture, induced pluripotent stem cells, organoids

## Abstract

Neurodegenerative diseases are progressive degenerative conditions characterized by the functional deterioration and ultimate loss of neurons. These incurable and debilitating diseases affect millions of people worldwide, and therefore represent a major global health challenge with severe implications for individuals and society. Recently, several neuroprotective drugs have failed in human clinical trials despite promising pre-clinical data, suggesting that conventional cell cultures and animal models cannot precisely replicate human pathophysiology. To bridge the gap between animal and human studies, three-dimensional cell culture models have been developed from human or animal cells, allowing the effects of new therapies to be predicted more accurately by closely replicating some aspects of the brain environment, mimicking neuronal and glial cell interactions, and incorporating the effects of blood flow. In this review, we discuss the relative merits of different cerebral models, from traditional cell cultures to the latest high-throughput three-dimensional systems. We discuss their advantages and disadvantages as well as their potential to investigate the complex mechanisms of human neurodegenerative diseases. We focus on *in vitro* models of the most frequent age-related neurodegenerative disorders, such as Parkinson’s disease, Alzheimer’s disease and prion disease, and on multiple sclerosis, a chronic inflammatory neurodegenerative disease affecting young adults.

## Introduction

Neurodegenerative diseases are age-related conditions characterized by uncontrolled neuronal death leading to a progressive decline in brain functions. These incurable and debilitating diseases are associated with a wide spectrum of clinical symptoms, including cognitive decline and/or the loss of locomotor functions. The number of affected individuals is growing due to the aging of human populations, and the severe effects of such diseases on the quality of life have increased the burden on healthcare systems worldwide (Heemels, 2016). Dementias in particular are responsible for the greatest burden of age-related neurodegenerative diseases. This is a broad term used to describe a number of conditions characterized by cognitive deficits, including Alzheimer’s disease (AD), vascular dementia, frontotemporal dementia, mixed dementia, and dementia with Lewy bodies. Other neurodegenerative diseases principally affect the locomotor system, including amyotrophic lateral sclerosis, Huntington’s disease, Parkinson’s disease (PD), multiple sclerosis (MS), and spinocerebellar ataxias.

The limited efficacy of drugs for the treatment of neurodegenerative diseases reflects their complex etiology and pathogenesis. In addition to aging, multiple risk factors contribute to susceptibility including environmental triggers and genetic factors. Therefore, more work is required to identify the underlying molecular mechanisms and corresponding pharmacological targets. In addition to the ethical concerns of animal experiments for medical research, the recent failure of several clinical trials targeting neurodegenerative diseases has raised doubts about the translatability of animal disease models to human patients, creating a demand for better research tools in this field (Olanow et al., 2008; Cummings et al., 2014; Schneider et al., 2014; Pfeuffer et al., 2016; Anderson et al., 2017). The development of novel *in vitro* models with greater physiological relevance may bridge the gap between current pre-clinical animal models and humans, allowing the discovery of promising drug targets that can be tested in future clinical trials. In addition, *in vitro* testing can reduce the duration and costs of translation by helping to identify the mechanism of action together with any associated risks.

Several *in vitro* approaches have been developed to understand the etiology and pathogenesis of a broad range of neurodegenerative diseases (Table 1) and we focus on those applied to PD, AD, prion diseases and MS in this review. In 1962 the first CNS organotypic culture was prepared from rat hypophysis tissue (Bousquet and Meunier, 1962). Cells derived from embryonic rat spinal cord and ganglia were subsequently cultured on collagen-coated glass, revealing their potential for organotypic differentiation and bioelectric properties suitable for electrophysiological studies (Crain, 1966). Since then, organotypic cultures have been prepared from brain slices encompassing several cerebral areas, including the hippocampus, substantia nigra, locus coeruleus, striatum, and basal forebrain (Lavail and Wolf, 1973; Whetsell and Schwarcz, 1983; Knopfel et al., 1989; Ostergaard et al., 1995; Robertson et al., 1997). Although tissue explants and organotypic slice cultures faithfully represent the cerebral architecture, they are difficult to prepare and maintain in a viable state, and their inherent variability leads to a lack of reproducibility in experiments (Walsh et al., 2005). The development of immortalized cell lines (Table 1) removed the need to use tissue as a source, but such cell lines often present genetic and metabolic abnormalities compared to normal human cells (Gordon et al., 2014). The advent of human embryonic stem cells (ESCs) and then human induced pluripotent stem cells (iPSCs) (Thomson et al., 1998; Takahashi et al., 2007) provided researchers with the tools to generate multiple differentiated cell types with the same genotype. Methods for the conversion of human somatic cells into iPSCs using retroviral transduction and transcription factors such as OCT4, SOX2, KLF4, and c-MYC have opened new frontiers in the development of *in vitro* disease models because iPSCs can be derived from patients, providing a source of neurons carrying the same genetic variants associated with pathogenesis in a defined microenvironment (Table 1). In the earliest experiments, iPSCs were cultured in undiversified 2D layers which were of limited value as disease models because they did not recreate authentic interactions between cells. To overcome this drawback, more sophisticated 3D culture models were developed, including spheroids, hydrogels, scaffolds derived from the extracellular matrix (ECM), and organ-like cultures (Fitzgerald et al., 2015). For instance, organoids preserve the cellular interactions that capture key structural and functional aspects of real organs at the micrometer to millimeter scale (Renner et al., 2017). Human brain organoids have recently emerged as invaluable tools to model the pathophysiology of diverse neurodegenerative diseases, facilitating a range of research applications including the analysis of disease mechanisms and progression, drug discovery, drug testing, and cell replacement therapy (Wang, 2018; Costamagna et al., 2019; Logan et al., 2019). The 2D and 3D models developed for the investigation of neurodegenerative diseases are summarized in Table 1.

**TABLE 1:** Cell culture systems that can be used to study neurodegenerative diseases (iPSC, induced pluripotent stem cell).

Neurodegenerative diseases	Immortalized cell lines	iPSC-derived cell lines	iPSCs (patient derived)	Organ-like model
**Parkinson’s disease**	Lotharius et al., 2005; Van Vliet et al., 2008; Zhang et al., 2014; Smirnova et al., 2016; Harischandra et al., 2019; Taylor-Whiteley et al., 2019	Devine et al., 2011; Nguyen et al., 2011; Ryan et al., 2013	Park et al., 2008; Soldner et al., 2009	Cavaliere et al., 2010; Daviaud et al., 2014; Son et al., 2017; Kim et al., 2019; Smits et al., 2019; Chlebanowska et al., 2020.
**Alzheimer’s disease**	Choi et al., 2014; Zollo et al., 2017	Kondo et al., 2013; Muratore et al., 2014; Sproul et al., 2014.	Yagi et al., 2011; Israel et al., 2012; Jang et al., 2012; Shi et al., 2012.	Lancaster et al., 2013; Kelava and Lancaster, 2016; Lee et al., 2016; Raja et al., 2016; Gonzalez et al., 2018; Jorfi et al., 2018.
**Creutzfeldt-Jakob disease**	Not available	Krejciova et al., 2017	Matamoros-Angles et al., 2018	Falsig and Aguzzi, 2008; Groveman et al., 2019.
**Multiple sclerosis**	Buntinx et al., 2003	Chen et al., 2013	Song et al., 2012; Douvaras et al., 2014; Di Ruscio et al., 2015.	Tan et al., 2018
**Amyotrophic lateral sclerosis**	Pansarasa et al., 2018	Chen et al., 2014; Fujimori et al., 2018	Dimos et al., 2008; Burkhardt et al., 2013; Sareen et al., 2013	Smith et al., 2015; Krencik et al., 2017; Osaki et al., 2018.
**Huntington’s disease**	Bidollari et al., 2018	Szlachcic et al., 2017	An et al., 2012; Camnasio et al., 2012; Juopperi et al., 2012; Nekrasov et al., 2016; Vigont et al., 2018; Mehta et al., 2018	Virlogeux et al., 2018
**Spinal muscular atrophy**	Not available	Not available	Ebert et al., 2009; Fuller et al., 2015; Zhang et al., 2017a; Valetdinova et al., 2019.	Hor et al., 2018
**Spinocerebellar ataxia**	Kumar D. et al., 2018; Maguire et al., 2019	Wang et al., 2015	Xia et al., 2013; Marthaler et al., 2016; Nayler et al., 2017; Sun et al., 2018; Chuang et al., 2019; Yang et al., 2019.	Not available
**Frontotemporal dementia**	Almeida et al., 2012	Not available	Zhang et al., 2013, 2017c; Ehrlich et al., 2015; Silva et al., 2016; Nakamura et al., 2019.	Seo et al., 2017

Neurons and glial cells (astrocytes, oligodendrocytes, and microglia) cultivated *in vitro* in static devices such as trans-well systems are useful tools for basic linear kinetic studies during drug discovery. However, the central nervous system (CNS) features structures such as the blood–brain barrier (BBB), which maintains homeostasis between the cerebral vasculature and the brain, and facilitates active interactions between the peripheral circulation and CNS. The BBB comprises specialized microvascular endothelial cells, pericytes, astrocytes and neurons that couple local neuronal functions to local cerebral blood flow and regulate the transport of blood components into and out of the CNS. Impairment of the BBB and the subsequent infiltration of peripheral immune cells through this barrier play important roles in the pathogenesis of several diseases (Liebner et al., 2018). In particular, immune cells exacerbate the pathology in AD patients and in related mouse models (Town et al., 2005; Baik et al., 2014; Zenaro et al., 2015; Pietronigro et al., 2019; Heneka, 2020; McManus and Heneka, 2020), and inflammation can modify or accelerate the progress of PD (Hirsch and Hunot, 2009; Sulzer et al., 2017). Thus, *in vitro* models should integrate brain organoids with BBB mimics in order to model neurodegenerative diseases more accurately. Furthermore, drug delivery to the CNS under physiological conditions is restricted by the BBB, so the integration of a functional vascular system into the organoid structure would facilitate the discovery of systemic drugs that target neurodegenerative disorders more effectively. In this review, we focus on recent advances in 3D culture systems for the investigation of neurodegenerative diseases, highlighting their strengths, weaknesses and potential future developments. Advances in these recent *in vitro* techniques will help us to understand the pathophysiology and underlying mechanisms of human neurodegenerative diseases, leading to the development of efficacious new therapies.

## Parkinson’s Disease

Parkinson’s disease is a movement disorder with a variable etiology. It is defined by deep gray matter volume loss caused by the destruction of neurons in the substantia nigra, leading to dopamine deficiency in the basal ganglia. PD affects 1–2% of individuals above the age of 65, and is the second most common age-related neurodegenerative disorder after AD (Van Den Eeden et al., 2003; Poewe et al., 2017). The hallmark of the disease is a progressive loss of nigrostriatal dopaminergic neurons, which normally present unmyelinated axons and form a large number of synapses (Bolam and Pissadaki, 2012; Kalia and Lang, 2015). This leads to several motor symptoms such as bradykinesia, rigidity, resting tremor, and postural instability. PD is also accompanied by a wide range of non-motor symptoms including sleep disturbance, constipation, dementia, cognitive decline, and olfactory deficits, which severely reduce the quality of life for PD patients (Obeso et al., 2010; Savica et al., 2010; Kalia and Lang, 2015). At the neuropathological level, PD is characterized by the accumulation of protein inclusions within the neuronal cell body and processes, known as Lewy bodies (LBs) and Lewy neurites, respectively (Spillantini et al., 1997). LBs are primarily composed of misfolded and insoluble aggregates of the presynaptic neuronal protein α-synuclein, but this protein also accumulates in other tissues (Kalia and Lang, 2015; Lazaro et al., 2017). Genetic forms of PD can provide information about the neuropathological mechanisms of the disease although they account for only 5–10% of all cases (Kalia and Lang, 2015). However, the molecular basis of neuronal degeneration in PD remains unclear, and current therapeutic strategies are limited to attenuating the motor symptoms (Charvin et al., 2018). Drug candidates that were moderately successful in pre-clinical studies have thus far failed to demonstrate efficacy in phase II or III trials, which is unsurprising given our incomplete knowledge of the pathophysiology and etiology of neurodegenerative diseases. Although patient stratification and the timing and duration of treatment are important factors, another key element required for successful drug development is the availability of robust pre-clinical screening tools for drug validation. Experimental tools such as *in vitro* 3D cell culture models are therefore needed to facilitate the selection of more promising lead compounds in order to eliminate failures at an earlier stage.

Several culture systems have been developed to study the pathogenesis of PD or to identify promising drug leads, each with advantages and limitations (Table 2). Traditional *in vitro* cell culture techniques usually involve 2D monolayers in standard tissue-culture plates, Petri dishes or cover slips. These are often based on immortalized cell lines such as human embryonic kidney 293 (HEK293) cells, human neuroglioma (H4) cells, or pheochromocytoma (PC12) cells derived from the rat adrenal medulla. The human neuroblastoma cell line SH-SY5Y is widely used in PD research because it reproduces the dopaminergic phenotype typical of PD pathology (Xicoy et al., 2017). A recent report demonstrated the formation of LB-like inclusions in SH-SY5Y cells cultured on a 3D matrix in serum-free Dulbecco’s modified Eagle’s medium (DMEM) for 7 days to allow differentiation, followed by exposure to recombinant human α-synuclein (Taylor-Whiteley et al., 2019). Although SH-SY5Y cells are widely used in PD research, their limitations include the lack of a standardized protocol to maintain them in culture, which leads to variable cell growth and inconsistent experimental outcomes (Buttiglione et al., 2007; Xicoy et al., 2017). Moreover, the survival of SH-SY5Y cells and their differentiation into neuron-like cells requires ECM proteins, neurotrophic factors and serum, and the use of different serum sources or concentrations can have a particularly significant effect on the experimental results (Encinas et al., 2000; Agholme et al., 2010). Depending on the protocol, SH-SY5Y cells can differentiate into various neuronal cell types in addition to dopaminergic cells, so it is necessary to verify the dopaminergic neuronal phenotype after differentiation by confirming the expression of dopamine and noradrenalin neurotransmitter transporters (Korecka et al., 2013; Xicoy et al., 2017). Given the lack of standardized cultivation methods, it is challenging to use SH-SY5Y-derived dopaminergic neurons for high-throughput cell-based screening assays even though the cells are easy to cultivate.

**TABLE 2:** Advantages and disadvantages of the different cell lines used to study Parkinson’s disease (iPSC, induced pluripotent stem cell).

Cell line	Type	Advantages	Disadvantages
**H4**	Human neuroglioma cells	Easy to culture and transfect.	Lacks dopaminergic phenotype.
**HEK 293**	Immortalized human embryonic kidney cells	Homogenous populations, suitable for large-scale experiments (Falkenburger and Schulz, 2006). Useful to study α-synuclein aggregation and mutations (Lazaro et al., 2014, 2016).	High passage numbers can lead to genetic and epigenetic alterations. Non-neuronal cell type (Falkenburger and Schulz, 2006).
**SH-SY5Y**	Human neuroblastoma cells	Differentiate into neuronal-like cells exhibiting cholinergic, dopaminergic, or noradrenergic phenotypes (Lopes et al., 2017)	Neuroblastoma origin may influence differentiation, viability, growth performance, metabolic properties and genomic stability. Multiple differentiation protocols lead to different outcomes (Xicoy et al., 2017).
**PC12**	Pheochromocytoma-derived cell line from the rat adrenal medulla	Synthesizes, releases and stores catecholamines. Easy to handle and homogeneous (Smirnova et al., 2016).	Not human. Derived from a neural tumor, which may alter signaling pathways.
**LUHMES**	Immortalized human embryonic mesencephalic cells	Already used in co-cultures with astrocytes (Efremova et al., 2017) and in a 3D culture system, prolonged survival after differentiation (Smirnova et al., 2016). More sensitive to toxins than other dopaminergic cell lines once differentiated (Tong et al., 2017).	Low transfection efficiency.
**Primary neurons**	Prepared from embryonic rodent brain tissue	Similar to human neurons in terms of morphology and physiology. Similar proliferation rate to human neurons. Suitable for the generation of genetic models (Lopes et al., 2017).	Ethical problems. Mixed culture. Variations among different culture preparations and difficult to maintain. Dissection procedure can introduce experimental variability (Xicoy et al., 2017). Species-dependent differences (Lopes et al., 2017).
**iPSCs**	Derived from patients	Capacity for self-renewal. Potential to differentiate into any cell type. Allow the generation of autologous pluripotent cells from any individual for disease modeling (Chlebanowska et al., 2020). Suitable for large-scale studies and personalized medicine (Kumar S. et al., 2018).	Challenging to identify disease-specific cell phenotypes that better represent pathogenesis. Do not mimic aging. Requirement of standardized protocols and quality controls to reduce technical variation. High costs (Kumar S. et al., 2018).
**Organoids**	Derived from patients	Provide a 3D environment of multiple cell types. Organized structure. Enhanced cellular maturity. Promising for screening compounds targeting the central nervous system. Possibility to study PD-related genes.	Highly variable. Need for improved vascularization and optimization of differentiation protocols. Time-consuming and expensive. Ethical problems (Marotta et al., 2020).

In contrast to SH-SY5Y cells, the Lund human mesencephalic (LUHMES) immortalized cell line provides a physiologically relevant system that is compatible with large-scale culture and achieves good batch-to-batch consistency (Zhang et al., 2014; Harischandra et al., 2019). LUHMES cells are derived from healthy 8-week-old human embryonic mesencephalic tissue and are immortalized by inserting the v-*myc* transgene under the control of a tetracycline-responsive promoter. These cells can be differentiated into mature dopaminergic neurons by adding cAMP, tetracycline and glial cell line-derived neurotrophic factor to the culture medium (Lotharius et al., 2005). LUHMES cells were used to develop a 3D model for neurotoxicity studies by applying the gyratory shaking technique, in which cells are shaken continually to encourage the formation of spherical aggregates containing astrocytes, neurons, oligodendrocytes and microglia (Van Vliet et al., 2008; Smirnova et al., 2016). The advantages of this system include (1) the ability of LUHMES cells to acquire a phenotype that is biochemically and morphologically similar to primary neurons, (2) the formation of a natural ECM that promotes cell–cell interactions, (3) the rapid maturation of the system, with astrocytes, neurons and oligodendrocytes undergoing both myelination and synaptogenesis within 25 days, and (4) the ability of LUHMES cell cultures to be maintained for 2 months while the cell populations continue to mature (Honegger, 2001; Van Vliet et al., 2008).

Primary cultures have the potential to overcome many of the difficulties inherent to cell lines, but isolating and culturing primary dopaminergic neurons from the post-mortem brains of adult/elderly patients is difficult. Therefore, primary dopaminergic neurons are usually obtained from embryonic murine brain tissue, particularly from the central midbrain area, because these cells differentiate rapidly in culture and form neurites and synapses (Gaven et al., 2014; Weinert et al., 2015). The primary neurons in this type of system are often contaminated with glial cells, but this can be regarded as an advantage. For example, this method has been exploited to study the therapeutic effect of microglial modulation in a mixed culture of primary neurons and microglia, thus showing how the microglia and the factors they release into the shared environment may affect neuronal function and survival (Che et al., 2018). Primary cortical neuron cultures have also been used to characterize the transport of synthetic α-synuclein fibrils (Freundt et al., 2012). The authors used a microfluidic device to separate neuronal cell bodies from their axons and from second-order neurons, and demonstrated that α-synuclein is internalized by neurons, can undergo both anterograde and retrograde transport, and can be released from primary neurons to be taken up by second-order neurons.

Although *in vitro* primary cultures have provided important clues about the mechanisms of PD and potential drug targets, they do not replicate the organization of cells and ECM within the CNS. More advanced systems, which are closer to *in vivo* animal disease models of PD, include organotypic cultures, 3D cultures and organoids (Chambers et al., 2009; Zhang et al., 2009; Shoji et al., 2016; Chlebanowska et al., 2020). An early example of a murine organotypic culture for PD research modeled nigrostriatal degeneration by cutting brains along the dorsoventral axis and culturing the slices containing cortex, corpus callosum, subventricular zone, striatum and substantia nigra, in order to ensure both the dopaminergic and glutamatergic pathways were represented in each slice (Cavaliere et al., 2010). Nigrostriatal pathway degeneration was induced by mechanical damage, in contrast to other models that used toxins such as 1-methyl-4-phenylpyridinium or 6-hydroxydopamine (Kearns et al., 2006). A more recent *ex vivo* culture system that models both the early and late stages of PD was prepared by the sagittal dissection of rat brains, allowing the simultaneous observation of cholinergic, GABAergic and dopaminergic neurons and the brain vasculature (Daviaud et al., 2014). Mechanical damage during slice preparation led to progressive degeneration of the nigrostriatal pathway, beginning with dopaminergic degeneration in the striatum and continuing, over the following weeks, with the degeneration of cholinergic and GABAergic neurons (Daviaud et al., 2014). Despite several advantages such as the replication of physiological processes and the well-controlled genetic background, these organotypic models are difficult to reproduce because the dissection procedures require high precision (Stahl et al., 2009). Moreover, the culture conditions required to maintain the brain tissue slices are difficult to standardize, and these models originate from animals with significant differences in neural anatomy, physiology, regulation, gene expression patterns, and drug metabolism compared to humans. The preparation of such primary cultures is expensive and time-consuming, requires significant expertise, and is not suitable for large-scale studies (Lopes et al., 2017).

To establish reproducible models more closely related to human pathophysiology, iPSC lines have recently emerged as one of the hottest and fastest moving topics in the life sciences. Many researchers have established PD-specific iPSC models by reprogramming somatic cells from PD patients. The first PD-specific iPSC line was derived from a patient affected by a sporadic form of the disease (Park et al., 2008). Since then, iPSC models of PD have been established from patients with susceptibility alleles in genes such as *LRRK2*, *PARKIN*, *SNCA*, *GBA*, and *PINK1*.

Mutations in the gene encoding leucine-rich-repeat kinase 2 (*LRRK2*) correlate with enteric inflammation and reinforce the role of peripheral inflammation in the initiation and/or progression of PD (Devos et al., 2013). Patient-derived iPSCs carrying the autosomal dominant G2019S mutation in the *LRRK2* gene have therefore been cultivated as 3D human neuro-ectodermal spheres (hNESs) and human intestinal organoids (hIOs) to study gene expression profiles linked to *LRRK2* in the neural and intestinal environments (Son et al., 2017). This revealed a broader alteration in gene expression profiles in the hIOs compared to hNESs, suggesting that *LRRK2*-G2019S may preferentially trigger the correlated intestinal symptoms of PD. An innovative protocol to generate neural tube lineages (including motor neurons and midbrain dopaminergic neurons) and neural crest lineages has been established using only small molecule neural precursor cells, which are robust, undergo immortal expansion, and do not require cumbersome manual culture and selection steps (Reinhardt et al., 2013). This protocol was recently combined with a microfluidics system to generate a 3D cell culture model based on neurons derived from human neuro-epithelial stem cells (hNESCs) from PD patients carrying the *LRRK2*-G2019S mutation (Bolognin et al., 2019). These authors generated a mixed population of neural cells that precisely captured the hallmarks of *LRRK2* pathogenesis, including degeneration, cell loss, and mitochondrial impairment. The results obtained using this *in vitro* system showed that the genetic background of PD patients can influence the degeneration of dopaminergic neurons, contributing to phenotypes other than that of the G2019S mutation itself. These data support the use of advanced *in vitro* models for future patient stratification and personalized drug development (Bolognin et al., 2019). The pioneering model consist in the differentiation of iPSCs into dopaminergic neurons inside a microfluidic bioreactor with a microtiter plate format. Within each bioreactor, cells embedded in Matrigel are loaded in the culture lane and perfused by medium flow. However, this system requires some improvements, including the use of alternatives to Matrigel in order to increase cell viability (Moreno et al., 2015). Recently, a microfluidic bioreactor was used to develop a high-throughput model that integrates laboratory automation technology, resulting in a robotic microfluidic cell culture system named *Pelican*. Specifically, this new system automates the cell culture protocols for hNESCs, allowing their experimentally reproducible differentiation into dopaminergic neurons. This platform may help to harmonize protocols between laboratories according to the standards of the SiLA consortium^1^ but it can also be adapted to different experimental conditions (Kane et al., 2019).

A 3D human midbrain organoid (hMO) populated with midbrain dopaminergic neurons (mDANs) derived from iPSCs has recently been described (Smits et al., 2019). The comparison of hMOs from healthy subjects and PD patients with the *LRRK2*-G2019S mutation revealed a reduction in the number and complexity of mDANs in the disease model, suggesting a neurodevelopmental defect in mDANs expressing *LRRK2*-G2019S. In addition to organoids derived from the genetic form of PD, the first organoid model of idiopathic PD was recently reported (Chlebanowska et al., 2020). The iPSCs were derived from the peripheral blood mononuclear cells of patients with idiopathic PD and were differentiated into large multicellular organoid-like structures. The authors observed statistical differences in the expression of early and late neuronal markers when comparing organoids prepared from PD patients and healthy volunteers, suggesting that hMOs have a remarkable potential for the investigation of PD pathogenesis *in vitro.* Overall, neurons derived from iPSCs and organoids from familial and idiopathic PD patients replicate the key pathological phenotypes of PD and are useful tools to study molecular pathways involved in the disease. They are also suitable for target validation, filling the gap between animal models and humans. Such 3D systems are necessary because they form complex structures that cannot be replicated in 2D models (Marotta et al., 2020). However, the application of such 3D models for large-scale studies and the generation of patient-specific organoids remains limited by the laborious procedures and high costs of preparation, and the need for written informed consent and approval from the ethics commission governing the institution conducting the experiments. This will restrict the practical application of patient-derived models for the foreseeable future.

## Alzheimer’s Disease

Alzheimer’s disease is a severe neurodegenerative disease characterized by the profound loss of cognitive functions and behavioral abilities, causing progressive deterioration in the patient’s quality of life. The pathogenesis of AD is incompletely understood (Scheltens et al., 2016). Two main forms of AD are recognized: early-onset familial Alzheimer’s disease (EOFAD) and late-onset Alzheimer’s disease (LOAD). EOFAD is relatively uncommon, accounting for less than 5% of all cases. It is diagnosed before the age 65, with an autosomal dominant pattern of inheritance reflecting the presence of one of 200 mutations discovered thus far in three key genes: amyloid precursor protein (*APP*), presenilin 1 (*PSEN1*), and presenilin 2 (*PSEN2*). LOAD is the most common form of AD, also known as sporadic AD, and typically occurs in people of age 65 or older without a family history of dementia (Bekris et al., 2010; Giri et al., 2016). Since 1992, the etiology and pathogenesis of AD has been explained by the amyloid cascade hypothesis, in which the accumulation of pathogenetic amyloid β (Aβ) protein, derived from APP, induces a vicious cycle that triggers the accumulation of neurofibrillary tangles (NFTs), neuronal cell death, and ultimately dementia (Reitz, 2012). The failure of anti-Aβ therapy, which was largely successful in mouse models of AD, helped to revise this hypothesis and define AD as a multifactorial disorder, also highlighting the limitations of animal disease models (Iqbal and Grundke-Iqbal, 2010). Animal models of AD proved to be inaccurate representations of human AD pathology, exclusively mimicking EOFAD (De Strooper, 2014; Henley et al., 2014). Indeed, animal models of AD tend to manifest only some pathological features, lacking important components such as extensive neuronal loss and the development of NFTs. One potential explanation is that rodent tau proteins may not be prone to aggregate formation due to differences in sequence and structure, together with the short life span of mice which does not allow sufficient time for the accumulation of events that take decades in humans (Laferla and Green, 2012). The development of sophisticated 3D *in vitro* models therefore provides a powerful complementary approach to overcome the limitations of current AD transgenic mice.

Many groups have developed 2D models based on iPSCs (Yagi et al., 2011; Israel et al., 2012; Mohamet et al., 2014; Hu et al., 2015; Moore et al., 2015; Lee et al., 2016; Li et al., 2016). In some cases, iPSCs were prepared from fibroblasts of EOFAD patients with mutations in *PSEN1* (A246E) or *PSEN2* (N141I), and neurons differentiated from the iPSCs were shown to secrete more Aβ42 than healthy matched controls, elevating the Aβ42 to Aβ40 ratio (Yagi et al., 2011). The reprogramming of fibroblasts from patients with familial and sporadic forms of AD replicated the significantly higher levels of Aβ40, active glycogen synthase kinase-3β (aGSK-3β) and hyperphosphorylated tau protein (Israel et al., 2012). The treatment of neurons derived from these AD patients with β-secretase inhibitors significantly reduced the levels of hyperphosphorylated tau and aGSK-3β, whereas a γ-secretase inhibitor showed no effects compared to controls, suggesting direct crosstalk between APP proteolytic processing during the activation of GSK-3β and tau phosphorylation in human neurons (Israel et al., 2012). CRISPR/Cas9 technology has been combined with iPSCs to generate knock-in human neurons carrying heterozygous or homozygous EOFAD mutations such as *APP*^*swe*^ and *PSEN1*^*M146V*^ (Paquet et al., 2016). Although these 2D models have revealed some of the key pathophysiological mechanisms of AD, they share with other 2D models the inability to reproduce all disease hallmarks, like any other model generated so far. For example, the levels of Aβ generated by these models were insufficient for the formation of plaques and related pathological features. Furthermore, the use of post-mitotic neurons prevented long-term cultivation and the *in vitro* models therefore could not replicate the age-dependent pathogenic events in the human AD brain (D’Avanzo et al., 2015). Most importantly, these 2D neuronal cultures lacked the supporting functions of glial cells, which play a key role in the pathogenesis of AD (Nagele et al., 2004; Gonzalez-Reyes et al., 2017).

The limitations of 2D cultures have encouraged the development of 3D models of AD, the first of which was based on an immortalized human neural stem cell line (ReN) containing mutations in the *APP* (K670N/M671L and V717I) and *PSEN1* (ΔE9) genes (Choi et al., 2014). These cells were found to accumulate both senile plaques and NFTs, two hallmarks of the disease not previously observed in 2D cultures or most animal models. Furthermore, ReN cells can differentiate into either neurons or glia, and the presence of Matrigel in the culture medium inhibits Aβ diffusion, promoting aggregation and plaque formation.

Cerebral organoids are the latest avenue for *in vitro* AD research, allowing the generation of organized structures similar to the human cortex. Human ReN cells were initially used to generate neurospheroids within arrays of microwells, and were found to produce both Aβ plaques and hyperphosphorylated tau protein after 8 weeks in culture (Jorfi et al., 2018). Another recent development is the use of vascularized human cortical organoids (vhCOs), which have the advantage to overcome the limitation of conventional organoids (these often feature necrotic regions due to limited oxygen and nutrient diffusion) by coupling cerebral organoids to a perfusion system that mimics the cortical vasculature (Kelava and Lancaster, 2016). This system is also superior to earlier brain organoids because vhCOs possess classical BBB properties such as the expression of tight junction proteins (ZO-1, occludin, and claudin-5) and nutrient transporters (P-glycoprotein and GLUT1), as well as high trans-endothelial electrical resistance. However, one disadvantage of vhCOs is that they lack blood cells within their vascular structures. In the near future, vhCOs may allow the screening of drugs that regulate the permeability of the BBB, thus providing an authentic model to study the regulation of interactions between the CNS and the periphery (e.g., the trafficking of Aβ and other molecules), as well as the mechanisms involved in the recruitment of leukocytes through the BBB during inflammation (Cakir et al., 2019).

To mimic blood flow, a triculture AD model including neurons, astrocytes and microglia was recently developed using a microfluidics platform (Park et al., 2018). These engineered human neural progenitor cells contained in a two-chamber microfluidic device replicated key features of AD, such as the aggregation of Aβ, the accumulation of hyperphosphorylated tau protein, and neuroinflammation. Interestingly, the authors demonstrated physiologically relevant interactions among neuronal cells, such as microglial recruitment, the release of pro-inflammatory cytokines and chemokines (CCL2, TNFα, and IFNγ) regulated by AD neurons and astrocytes, and microglial neurotoxic activation contributing to neuron/astrocyte damage. In addition to glia, the brain also contains many other non-neuronal cell types that ensure the long-term survival and function of neurons (Zhao et al., 2015). For example, the BBB blocks the passage of many cells and molecules between the CNS and peripheral blood circulation, and oligodendrocytes insulate neuronal axons and promote rapid axonal transmission. However, the contribution of each of these non-neuronal cell types to AD-associated neurodegeneration is not fully understood. A complex 3D model of neural cell culture in a microfluidic system was recently developed by adding brain endothelial cells with a BBB-like phenotype (Shin et al., 2019). This AD platform simulates the cerebral–vascular interface, successfully mimicking several of the vascular phenotypes observed in AD patients, including the greater permeability of the BBB coincident with the downregulation of certain tight junction proteins (claudin-1 and claudin-5), adherens junction proteins and VE-cadherin, as well as the upregulation of matrix-metalloproteinase-2 (MMP-2), the accumulation of reactive oxygen species (ROS), and the aggregation of Aβ on the abluminal side of the BBB endothelium. This experimental system may therefore reveal the physiological and pathological mechanisms of BBB dysfunction in AD and could be used as a standardized drug-screening platform.

As discussed above for PD models, patient-derived iPSCs have also been used to model AD. The first was a 3D neurospheroid model based on iPSCs derived from the peripheral leukocytes of five patients with sporadic AD (Lee et al., 2016). The authors demonstrated that the administration of BACE1 or γ-secretase inhibited the formation of pathological Aβ peptides. Interestingly, the use of 3D neurospheroids highlighted individual variations in the efficacy of BACE1 that were related to differences in individual genetic backgrounds (APOE genotypes). This 3D model not only provided the basis for more accurate drug-screening methods, but facilitated subsequent studies of individual phenotype variations thus allowing a personalized approach for the treatment of AD (Lee et al., 2016). Recently, a physiological 3D model of AD was described in which neural areas with a cortical-like organization were generated from fibroblast-derived iPSCs donated by adult EOFAD and Down syndrome patients (Gonzalez et al., 2018). These highly reproducible cerebral organoids spontaneously accumulated aggregates of Aβ and hyperphosphorylated tau protein. The analysis of caspase-3 activation indicated a rate of neuronal death proportional to the accumulation of protein aggregates, suggesting that cerebral organoid cultures develop certain neurodegenerative features in common with the AD brain (Gonzalez et al., 2018). Nevertheless, these methods are still limited by their inability to model more complex events including cell-cell interactions and migration in the developing brain, and future research should focus on improvements that accommodate such features.

Oligodendrocyte dysfunction and the loss of white matter during the progression of AD are key features of human patients and rodent models, probably contributing to the neuronal degeneration (Desai et al., 2010; Bartzokis, 2011). The differentiation of iPSCs can be used to produce oligodendrocytes, and recently this has been exploited to generate oligocortical spheroids for the analysis of myelination in the CNS (Hu et al., 2009; Ehrlich et al., 2017; Hubler et al., 2018; Madhavan et al., 2018; Marton et al., 2019). Moreover, neurons do not establish mature synaptic connections in 3D culture models (Huch et al., 2017) and the proportion of cells differs from that in the human brain, mainly comprising neurons with few glial cells and no oligodendrocytes. These sophisticated *in vitro* culture methods should therefore be improved to represent the cell population found *in vivo*, which will in turn make it easier to identify the pathological mechanisms underlying AD. The use of AD mini-brains offers the possibility of validating disease mechanisms and should lead to the identification of new pathways contributing to the progression of AD.

## Prion Diseases

The hallmark of several age-related neurodegenerative diseases is the formation and aggregation of misfolded proteins in the CNS. PD and AD share key biophysical and biochemical characteristics with prion diseases, a group of neurodegenerative diseases triggered by the misfolding of the cellular prion-related protein (PrP^*C*^). The molecular mechanism underlying these diseases is the conformational misfolding of monomeric PrP^*C*^ to form protein aggregates known as scrapie prion proteins (PrP^*Sc*^) or proteinaceous infectious particles. The abnormally folded protein is protease resistant and rich in β-strands, enabling the formation of oligomer/fibril structures that are involved in three pathological forms of disease: the sporadic form, also called Creutzfeldt-Jakob disease (CJD), the genetic form, and the acquired form. The genetic forms of prion diseases are caused by mutations in the prion protein gene (*PRNP*) that favor the abnormal folding events discussed above and are classified as genetic CJD, Gerstmann-Sträussler-Scheinker syndrome, and fatal familial insomnia. The acquired forms of are known as variant CJD (vCJD), iatrogenic CJD (iCJD), and kuru (Collins et al., 2001; Geschwind, 2015; Scheckel and Aguzzi, 2018). However, the most common form affecting humans is sporadic CJD, which accounts for 80–95% of cases. The peculiarity of these diseases is the seeding property of PrP^*Sc*^, which is able to convert a normal PrP^*C*^ into another copy of PrP^*Sc*^ with the same seeding capability, leading to the exponential formation of prions (Geschwind, 2015). The pathological hallmarks of prion diseases include neuronal loss, the activation of microglia and astrocytes, spongiform changes, and the formation of PrP^*Sc*^ aggregates and deposits. The pathogenesis of prion diseases is not fully understood and diagnosis is possible only when the disease has already reached an advanced stage (Knight, 2008).

The development of 3D cell culture models based on the differentiation of human iPSCs into neuronal tissue has produced a range of high-throughput platforms to test infection methods and treatments, and to address fundamental questions in prion biology. PrP-expressing human and animal cell lines in 2D culture generally do not establish stable prion infections and do not display cytopathic signs. The first suitable model was based on murine cerebellar organotypic slices, which revealed the amplification of PrP^*Sc*^ following exposure to prions (Falsig and Aguzzi, 2008). Improved models have been developed based on murine neural stem cells, early examples of which included 3D neurospheroids composed of neurons and astrocytes (Collins and Haigh, 2017). The neurospheroids reached maturity after 10 days and could be maintained in culture for up to 1 month. Prions were able to spread and induce toxic changes in the neurospheroids during growth and after differentiation, allowing the investigation of underlying mechanisms (Collins and Haigh, 2017). Despite the advantages of this model, the lack of microglia may influence the experimental outcome. Indeed, prion diseases are normally associated with astrogliosis, which also involves the recruitment of microglia, and their absence or depletion may influence the growth rate of astrocytes (Marella and Chabry, 2004).

Recently, cerebral organoids derived from human iPSCs were established for the first time as a 3D model to study genetic prion diseases in individuals with a predisposition caused by the mutation *PRNP*-Y218N (Matamoros-Angles et al., 2018). Although the cultures were characterized by astrogliosis and tau hyperphosphorylation, this model failed to replicate PrP^*Sc*^ accumulation, and neurons generated from human iPSCs with or without the Y218N mutation were unable to propagate infection following inoculation with human PrP^*Sc*^ from either spontaneous CJD or *PRNP*-Y218N individuals (Matamoros-Angles et al., 2018). To replicate human prion infection and pathogenesis, cerebral organoids were inoculated with brain homogenates from post-mortem samples of patients affected by different forms of spontaneous CJD, revealing that seeding activity was influenced by the spontaneous CJD subtype (Groveman et al., 2019). This new *in vitro* 3D model for the investigation of human prion disease provides insight into the pathological events caused by different human prion subtypes, and offers a promising platform for drug discovery using a relevant human tissue background. Although cerebral organoids can be maintained in culture for a long time, their limitations include structural heterogeneity, the lack of vascularization, and the absence of non-neuronal cells such as endothelial cells and microglia.

## Multiple Sclerosis

Multiple sclerosis is the most common chronic inflammatory, demyelinating and neurodegenerative disease of the CNS in young adults, with an onset at 20–40 years of age and a higher prevalence in women (Compston and Coles, 2008). MS neuropathology involves the appearance of focal plaques containing demyelinated axons, proliferating astrocytes, activated microglia, infiltrating lymphocytes and macrophages, and a reduced population of oligodendrocytes, typically located around post-capillary venules characterized by a breakdown of the BBB (Mahad et al., 2015; Huang et al., 2017; Stys and Tsutsui, 2019). Clinical symptoms of MS include motor dysfunction, fatigue, tremor, nystagmus, acute paralysis, loss of coordination or balance, numbness, disturbed speech and vision, and cognitive impairment (Ghasemi et al., 2017). The disease usually begins with a primary relapsing-remitting phase (RRMS) during which the symptoms are intermittent. Over the next 10–15 years, this transitions to a secondary progressive phase (SPMS) with continuous deterioration. But ∼15% of cases are classed as primary progressive (PPMS), in which the disease progression is relentless from the onset (Mahad et al., 2015; Huang et al., 2017). MS is a heterogeneous, multifactorial, immune-mediated disease that is influenced by both genetic and environmental factors. Two main hypotheses have been proposed for the pathogenetic mechanism: (1) the “outside-in” hypothesis, mostly based on findings from experimental animal models of MS, states that the CNS is invaded by auto-reactive T cells activated in the periphery, contributing to inflammation, BBB leakage and tissue damage (Lucchinetti et al., 2000; Frohman et al., 2006); and (2) the “inside-out” hypothesis states that MS is a primary degenerative disease but its severity increases following an amplification of the immune response (Henderson et al., 2009). In the latter case, the trigger may be a primary defect in the oligodendrocytes, such as a mutation that causes them to die off, resulting in the activation of microglial cells (Hemmer et al., 2015). Drugs that promote the remyelination of damaged axons are required to overcome the neurodegenerative phase of MS. However, the molecular and cellular basis of the myelin repair deficiency during the progression of MS are still unclear.

Given the complexity of the neurodegenerative mechanism during the progression of MS, rodent 3D organotypic brain slices have been widely used to study pathogenesis because they contain different regions of the CNS, allowing experimental manipulations that cannot be easily made *in vivo*. Organotypic brain slices can be maintained *ex vivo* for several months and allow researchers to study the physiological process of myelination in a system where complex cell–cell relationships are preserved (Schnadelbach et al., 2001). The tissue slices are often placed on porous membranes in cell culture dishes and cultured at an air–liquid interface, with the medium in contact with the permeable membrane (Gogolla et al., 2006). The slices can be demyelinated *in vitro* by applying toxins or an immune challenge, but undergo spontaneous remyelination (Birgbauer et al., 2004; Zhang et al., 2017b). This *in vitro* system can also be used to screen promising drugs that promote remyelination (Zhang et al., 2011; Doussau et al., 2017; Tan et al., 2018).

Although rodent brain slices have been widely used in MS research, several compounds that promote remyelination and neuronal survival in rodent models of MS have failed when tested in human clinical trials, highlighting the inability of animal models to completely replicate the complexities of the human disease phenotype (t Hart et al., 2011). As described above for PD, AD and prion diseases, new *in vitro* tools based on 3D cultures are therefore required to replicate the pathophysiology of human MS and identify the most promising drug targets. Culture systems based on human oligodendrocytes or oligodendrocyte precursor cells (OPCs) have been developed to address the deficiencies of animal MS models. These are preferred because they have the ability to differentiate and generate myelin *in vitro* in the absence of signals from axons (Bechler et al., 2015). Protocols based on OPCs have been particularly successful because they can generate mature myelinating oligodendrocytes (Dugas and Emery, 2013; Barateiro and Fernandes, 2014; Chew et al., 2014). Mouse, rat and human OPCs differ in terms of their longevity in culture, with rat OPCs proving the easiest to isolate and maintain whereas human OPCs take the longest to develop the classical mature oligodendrocyte phenotype. Moreover, human OPCs must be isolated from brain biopsies, with the attendant issues surrounding ethical clearance. For these reasons, human oligodendrocyte cultures are usually initiated using OPCs generated *de novo* from the differentiation of human embryonic stem cells, iPSCs (Hu et al., 2009), fetal cortical neurospheroid-derived cells, or umbilical cord-derived stem cells (Chen et al., 2013; Leite et al., 2014).

The use of stem cells raises the possibility that 3D cultures could be generated from the cells of patients carrying specific MS susceptibility alleles, allowing the analysis of genetic mechanisms and the development of personalized therapies. Dermal fibroblasts from a 35-year-old patient affected by RRMS were reprogrammed; these were named MS iPSCs and were successfully differentiated into mature astrocytes, oligodendrocytes and neurons with normal karyotypes, although MS iPSC-derived neurons showed atypical electrophysiological characteristics (Song et al., 2012). In addition, iPSCs from PPMS patients of both sexes in the age range 50–62 years have been used to generate OPCs that achieved the efficient myelination of neurons *in vivo* and could be suitable for the development of autologous cell-replacement therapies for MS in the future (Douvaras et al., 2014). The advent of iPSC-derived OPCs allows researchers to study the impact of genetic variants on specific cell populations to facilitate the identification of disease subtypes and the functional evaluation of genetic variants and their role in MS (Di Ruscio et al., 2015). MS is a complex, multifactorial disease with multiple susceptibility phenotypes, many of which are related to the adaptive immune system (International Multiple Sclerosis Genetics Consortium [IMSGC] et al., 2011; Stys and Tsutsui, 2019). A broad panel of patients and controls is therefore required to generate a sufficient number of cell lines for the detection and validation of biologically relevant MS phenotypes (Song et al., 2012). The real limitation of MS organotypic models, such as iPSC-derived OPC cultures, is that they exclude the peripheral immune system, which is a key player in both the progression of MS and regeneration. It is unclear whether neuronal damage reflects the different phenotypes of autoreactive T lymphocytes. Therefore, it may be possible in the future to establish co-cultures of neurons and T cells derived from individual MS patients. This would allow both the detection of genetic variants in the T cells involved in neuronal damage and the screening of neuroprotective compounds blocking the detrimental effects of this T cell subpopulation, in order to improve our understanding of their role in the progression of MS.

## Conclusion and Future Directions

Cellular models of neurodegenerative diseases range in complexity from conventional monolayers derived from immortalized cell lines to complex multicellular 3D tissue mimetics based on patient-derived iPSCs, which can replicate many disease hallmarks and *in vivo* physiological conditions, such as protein aggregation. Indeed, 3D cell culture systems can recapitulate the extracellular aggregation of Aβ and NFT typical of AD (Choi et al., 2016) and can spread the PrP^*Sc*^ responsible for prion diseases (Falsig and Aguzzi, 2008; Collins and Haigh, 2017). Furthermore, the aggregation of α-synuclein has been demonstrated in neurons differentiated from iPSCs derived from sporadic and familial PD patients, suggesting they can reproduce this hallmark of PD without the use of exogenous factors (Oh, 2019).

Traditional cell culture systems and animal models have improved our understanding of human neurodegenerative diseases, but models based on recent technological innovations in 3D culture systems can achieve the better characterization of pathological mechanisms, which also makes them more suitable for high-throughput drug screening. Indeed, the advent of new technologies involving the generation of organoids from iPSCs and the use of microfluidic devices could accelerate drug discovery compared to current approaches based on animal models. Despite these recent advances, we still lack a 3D model that recapitulates all the key aspects of neurodegenerative diseases and thus allows the detailed analysis of their pathophysiology.

Most *in vitro* cultures are composed primarily of neuronal cells, and their complexity (and therefore suitability as disease models) should be increased by adding microglial cells, astrocytes, oligodendrocytes, and pericytes to better mimic the structural and molecular complexity of the CNS environment in different diseases (Ormel et al., 2018; Song et al., 2019; Speicher et al., 2019). The genetic background of the cells used to establish the model may also influence drug responses, as recently shown for iPSCs isolated from PD patients and cultured in a microfluidic 3D platform (Bolognin et al., 2019). The unique genetic background of each individual can affect their susceptibility to neurodegenerative diseases. Patient-derived iPSCs could therefore reveal common pathways even in sporadic disease cases, allowing the development and testing of new therapeutic strategies. Interestingly, iPSC-derived neurons from patients with sporadic AD featured mutations that have not been identified by genome-wide association studies, and these mutations generated neuronal phenotypes in a manner analogous to familial AD, suggesting common pathogenetic mechanisms (Israel et al., 2012). However, previous experience with the handling of iPSCs has revealed the potential for *in vitro* reprogramming, which is a significant drawback. Polymorphisms affecting neuronal functions could overcome defects introduced during reprogramming process or generated by genomic instability due to the long *in vitro* culture durations. Furthermore, donor age, sex, and ancestry could influence the physiology of the iPSCs (Mackey et al., 2018). X-linked genes in cells generated from female patients with neurological diseases (including MS) tend to be inactivated, leading to a significant effect in the neurodegeneration models (Mekhoubad et al., 2012). Moreover, skin fibroblasts from older donors show very inefficient iPSC reprogramming (Ohmine et al., 2012), so we must also take into account the fact that neurodegenerative diseases are often aged related, or (in the case of MS) diagnosed later in life. Fibroblasts were recently shown to possess anatomic positional memory, which is clearly important for reprogramming strategies (Sacco et al., 2019). Moreover, in aged-associated diseases such as PD and AD, neuronal cells suffer epigenetic drift, mitochondrial dysfunction and the accumulation of damaged proteins, which can disrupt gene regulation and homeostasis leading to abnormal phenotypes. These features could be lost by cell rejuvenation during the reprogramming of iPSCs, although this could be addressed by the direct conversion of aging neurons (Mertens et al., 2018). It therefore follows that the variability, together with the inadequate purity of the reprogrammed cells and the lack of standardized methodologies still hamper the reproducibility of iPSCs-derived methodologies across different laboratories.

**FIGURE 1:**
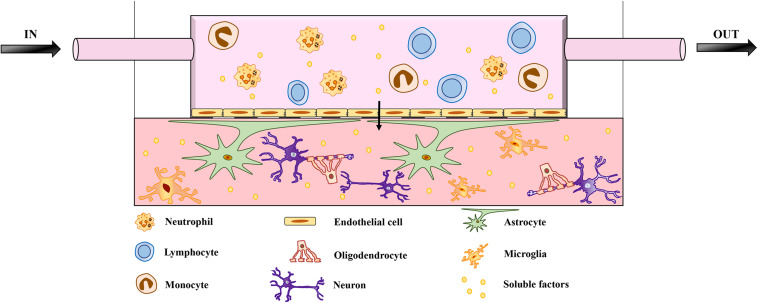
Schematic representation of microfluidic brain model. The model features neurons and glial cells embedded in a matrix. The architecture includes a flow of medium mimicking the BBB, enriched with soluble factors and peripheral immune cells, which are key players in neuroinflammation and neurodegeneration. The migration of peripheral immune cells through the BBB has been implicated in the pathogenesis of several neurodegenerative diseases. The role of infiltrating peripheral immune cells has been investigated in detail for MS, which involves the breakdown of the BBB and multifocal inflammation caused by the innate and adaptive immune systems. However, BBB impairment and the infiltration of peripheral immune cells also correlate with the pathogenesis of other neurodegenerative diseases, such as AD and PD. Adding a fluidic system to mimic the BBB is therefore necessary to investigate the pathological mechanisms of neurodegenerative diseases and eventually to study the transport of drugs across the BBB.

Brain function and homeostasis depend not only on neuronal cells but also on the regulation of cerebral blood flow, which supplies nutrients and oxygen while removing waste products. The BBB is essential in this context because it connects the CNS to both the peripheral circulation and the immune system (Zhao et al., 2015). The disruption of the BBB followed by the infiltration of peripheral immune cells is correlated with the pathogenesis of neurodegenerative diseases such as AD and PD (Schwartz and Shechter, 2010; Poewe et al., 2017; Zenaro et al., 2017). Complex *in vitro* models that include fluidic systems to mimic the BBB are therefore required to fully replicate neurodegenerative diseases affecting the CNS (Aucouturier et al., 2000; Zlokovic, 2008; Lopes Pinheiro et al., 2016). Recent studies have included vascularization in 3D models, highlighting the importance of this component (Lee et al., 2017; Mansour et al., 2018; Pham et al., 2018; Cakir et al., 2019; Worsdorfer et al., 2019). The most recent BBB model uses a chip to mimic the physiological interactions between brain endothelial cells, pericytes, and astrocytes, although the neuronal component and immune cells are still missing (Ahn et al., 2020). The role of immune dysfunction in neurodegeneration can only be understood in detail by the incorporation of immune cells into future 3D models (Figure 1). Indeed, an unlimited source of circulating leukocytes can be obtained from human blood, and these cells should therefore be considered in future models of neurodegenerative diseases. A recent study using a microfluidic system to mimic central–peripheral innate immunity in AD (Park et al., 2019) suggested that targeting the crosstalk between central and peripheral immune cells may reduce the immunological burden in other neuroinflammatory diseases of the CNS. Moreover, the BBB should be considered as a key component of 3D brain models because it is one of the major challenges in the development of drugs targeting the CNS (Pandit et al., 2019). The BBB prevents up to 98% of small-molecule drugs from reaching the brain (Pardridge, 2005; Neuwelt et al., 2008). The delivery of drugs to the CNS therefore requires new strategies that take advantage of BBB transporter systems, highlighting the need for more research into the physiology this barrier.

Although *in vitro* models of neurodegenerative diseases are still incomplete, the 3D culture methods discussed herein offer an important new strategy to characterize disease mechanisms, leading to the discovery of new therapies. Personalized treatments are not yet economically viable, but 3D models based on patient-derived cells will show how the genetic landscape of the human population contributes to the pathogenesis of neurodegenerative diseases, bringing the prospect of personalized medicine another step closer.

## Author Contributions

AS and GI searched the literature, performed review design, designed the figure and tables, and wrote the manuscript. BR, EZ, and GC contributed to review conception and manuscript revision.

## Conflict of Interest

The authors declare that the research was conducted in the absence of any commercial or financial relationships that could be construed as a potential conflict of interest.

## Footnotes

1 https://sila-standard.com/
